# Notes and News

**Published:** 1893-05-27

**Authors:** 


### Notes and News.

COLLECTED AND COMMUNICATED.

We are informed by a correspondent of an ingenious method adopted by a Board of Guardians to keep a woman from the public-house. The woman's husband contracted small-pox, whereupon the Board paid her to nurse her husband. Our correspondent expresses shocked surprise at such a proceeding. If the woman was in the habit of frequenting the public-house, we cannot but regard the method by which the Guardians obtained a hold on her by paying her, with the probability of thus preventing the spread of the disease, as more commendable than otherwise.

A gentleman, signing H. T. Edge, F.T.S., of 19, Avenue Road, Regent's Park, writes in reference to a notice in our columns of an article in the *Nineteenth Century*: "I notice that in a paragraph in your issue for May 13th you refer to an alleged exposure of Madame Blavatsky by Professor Max Müller in the *Nineteenth Century.* If this exposure rests in the fact that in some of her writings Sanskrit and Greek words are wrongly spelt, or transliterated according to rules different from those of the Professor, it will be sufficient to say that Madame Blavatsky never made any claim to scholarship, and that hence, Max Müller, in exposing a pretension she never made, is simply beating the air. A perusal of the article, however, shows that Madame Blavatsky has run counter to him by interpreting the Buddhist Scriptures in the light of the *Esoteric Philosophy,* instead of in the light of Western scholarship, and that he consequently feels obliged to defend his point of view by throwing discredit on hers. As, however, he in one place denies the existence of any esoteric teachings in Buddhism, and in another asserts it, it is clear that he cannot have any decided opinion on the subject. All the same, though Max Müller knows very little of the inner meaning or Oriental systems, Theosophists have no wish to deny their obligations to him as an able translator."

The first annual meeting and dinner of the London and Counties Medical Protection Society (Limited), will be held at the Frascati Restaurant, Oxford Street, London, on Thursday, June 15th.

Thoughts are now frequently turning towards holiday times. It may be useful to many of our readers to know that Messrs. George Phillip and Son, 32, Fleet Street, make a speciality of literature of all kinds, especially useful to tourists. Those who send for catalogues can obtain useful information for all kinds of travelling by river, rail, cycle, and for walking tours abroad and at home.

The Chairman and Committee of Management of the Royal Westminster Ophthalmic Hospital make a special appeal for funds towards rebuilding the hospital. The accommodation in the present building both for out and in-patients is wholly inadequate to the daily increasing claims for relief. To erect a suitable structure on a larger site £50,000 is needed. Subscriptions can be sent to Messrs. Coutts and Co., 59, Strand, or to the Secretary of the hospital.

A special appeal on behalf of the Royal South London Ophthalmic Hospital, Southwark, shows that a larger assured income is a pressing want of the institution. The forty beds and large out-patients' department cannot be maintained at a less cost than £3,000 per annum. The wants of the very poor population in which the hospital is situated appeal to those who are better off in other localities. His Royal Highness the Prince of Wales has shown his appreciation of the good work of the hospital and of the need for it by becoming an annual subscriber of £10 10s.

In connection with the Hospital Saturday Fund, a meeting of the Council appointed to organise cycling and athletic sports in aid of the medical charities associated with the Hospital Saturday Fund, was held recently. The Hon. Secretary reported that the Executive Committee had arranged to carry out a cycling meeting at Herne Hill on May 27th, and an athletic meeting at Paddington on August 26th. The Lord Mayor and Sheriffs had kindly agreed to be present at Herne Hill on May 27th. Letters were read from secretaries of several clubs, who are enthusiastically working in the interests of these sports, and the Committee now appeal to all club officials to do their utmost to circulate tickets widely in order to make the meeting worthy of metropolitan cyclists.

The *Watford Observer* publishes some correspondence between the Honorary Secretary of the Cottage Hospital and Dr. C. Deun Christmas, who admitted a child suffering from diphtheria to the hospital contrary to the rules. Dr. Christmas contends that it is justifiable to admit these cases to the wards of general hospitals, and we must assume, therefore, that he is unaware that the Act of 1890 was specially passed to prevent the dangers arising from this practice. The Committee have, quite rightly, resolved that no cases shall in future be admitted by anybody, and we regret to see the line taken by Dr. Christmas, as it is opposed to the general well-being, and contrary to the teachings of experience. Any Committee would be censurable which permitted cases of diphtheria to be treated in the wards of a general hospital. We regret to hear that the Matron, Miss Hillyer, has contracted diphtheria, but we hope she will make a rapid and complete recovery.

